# Supplementary material for: Identification of photoperiod-induced specific miRNAs in the adrenal glands of Sunite sheep (Ovis aries)
Source: Front Vet Sci. 2022 Jul 22;9:888207. doi: 10.3389/fvets.2022.888207 (PMC9354845; doi:10.3389/fvets.2022.888207)
Supplement: Supplementary file 1 [file Data_Sheet_1.ZIP › Supplementary file/miRDeep2 file.zip!/miRDeep2 file/Know_Structure/LP42A1/oar-mir-143.pdf]

miRBase precursor : oar-mir-143
Total read count : 390107
oar-miR-143 read count : 387121
remaining reads : 2986

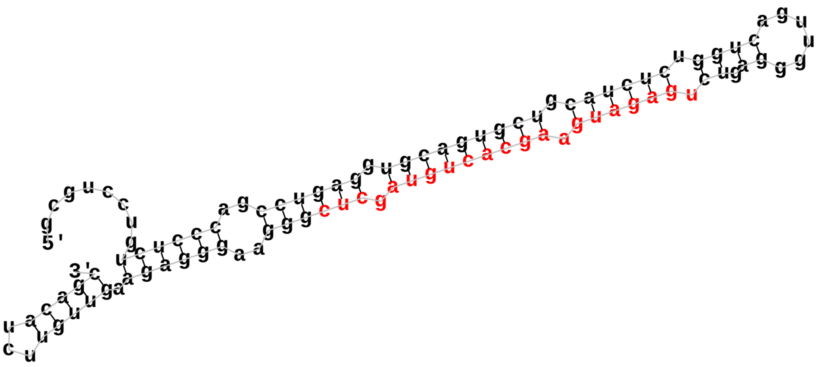

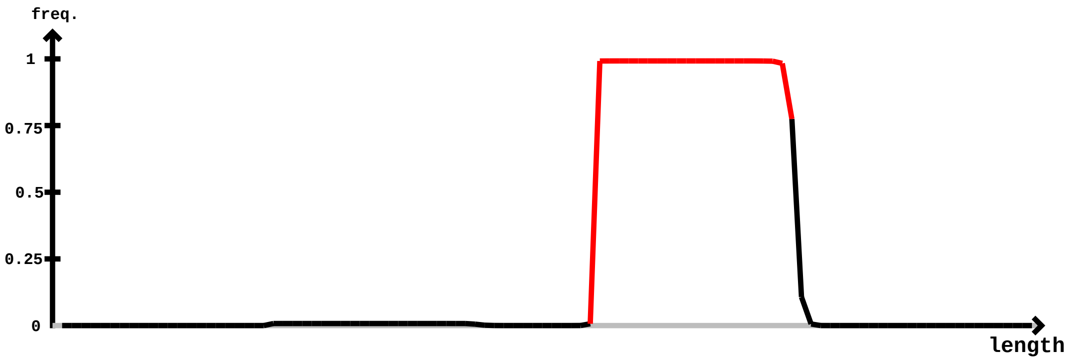

oar-miR-143

```
5'- gcguccugucucccagccugaggugcagugcugcaucucuggucaguugggagucugagaugaagcacuguagcucgggaagggagaaguuguucuacagc  -3'    exp
    ........((((((..((((((.(((((((((.((((((.((((......)).)).)))))).))))))))).))))))..)))))).(((((...)))))   reads     mm        sample
    ....................aggugcagugcugcaucucug............................................................   1         0         seq
    ....................aggugcagugcugcaucucugg...........................................................   3         0         seq
    ....................aggugcagugcugcaucucuggu..........................................................   3         0         seq
    .....................ggugcagugcugcaucucu.............................................................   5         0         seq
    .....................ggugAagugcugcaucucu.............................................................   2         1         seq
    .....................ggugcagugcugcaucucug............................................................   11        0         seq
    .....................ggugcagugcugcaucucugg...........................................................   912       0         seq
    .....................ggugcagugcugcaucucuggu..........................................................   1495      0         seq
    .....................ggugcagugcugcaucucugguc.........................................................   475       0         seq
    .....................ggugcagugcugcaucucugguca........................................................   72        0         seq
    .....................ggugcagugcugcaucucuggucagu......................................................   1         0         seq
    .....................ggugcagugcugcaucucuggucaguu.....................................................   2         0         seq
    ......................gugcagugcugcaucucugguc.........................................................   3         0         seq
    ....................................................gucugagaugaagcacuguagc...........................   1         0         seq
    .....................................................ucugagaugaagcacuguagc...........................   6         0         seq
    .....................................................ucugagaugaagcacuguagcu..........................   9         0         seq
    .....................................................ucugagaugaagcacuguagcuc.........................   76        0         seq
    ......................................................cugGgaugaagcacugu..............................   1         1         seq
    ......................................................cugagaugaagcacugu..............................   1         0         seq
    ......................................................cugagaugaagcacuguag............................   6         0         seq
    ......................................................cugagaugaagcacuguagc...........................   42        0         seq
    ......................................................cugagaugaagcacuguagcu..........................   630       0         seq
    ......................................................cugagaugaagcacuguagcuc.........................   1771      0         seq
    ......................................................cugagaugaagcacuguagcucg........................   116       0         seq
    ......................................................cugagaugaagcacuguagcucgg.......................   2         0         seq
    .......................................................ugagaugaagcGcugua.............................   2         1         seq
    .......................................................ugagaugaagcaUugua.............................   1         1         seq
    .......................................................ugagaugaagcacugua.............................   137       0         seq
    .......................................................ugagaugaagcacuguU.............................   3         1         seq
    .......................................................ugagaugaagcacuguG.............................   2         1         seq
    .......................................................ugagaugaagUacugua.............................   2         1         seq
    .......................................................ugagGugaagcacugua.............................   1         1         seq
    .......................................................ugaCaugaagcacugua.............................   3         1         seq
    .......................................................ugagaugaagcacuguag............................   229       0         seq
    .......................................................ugagaugaagcacuguagc...........................   3168      0         seq
```

oar-miR-143

```
gcguccugucucccagccugaggugcagugcugcaucucuggucaguugggagucugagaugaagcacuguagcucgggaagggagaaguuguucuacagc
.......................................................ugagaugaagcacuguagcu..........................   80779    0    seq
.......................................................ugagaugaagcacuguagcuc.........................   258366   0    seq
.......................................................ugagaugaagcacuguagcucg........................   39369    0    seq
.......................................................ugagaugaagcacuguagcucgg.......................   2234     0    seq
.......................................................ugagaugaagcacuguagcucggg......................   18       0    seq
........................................................gagaugaagcacuguagc...........................   3        0    seq
........................................................gagaugaagcacuguagcu..........................   16       0    seq
........................................................gagaugaagcacuguagcuc.........................   70       0    seq
........................................................gagaugaagcacuguagcucg........................   18       0    seq
.........................................................agaugaagcacuguagcuc.........................   2        0    seq
.........................................................agaugaagcacuguagcucgg.......................   19       0    seq
.........................................................agaugaagcacuguagcucggg......................   3        0    seq
..........................................................gaugaagcacuguagcu..........................   1        0    seq
..........................................................gaugaagcacuguagcucgg.......................   5        0    seq
..........................................................gaugaagcacuguagcucggg......................   4        0    seq
..........................................................gaugaagcacuguagcucggga.....................   3        0    seq
..........................................................gaugaagcacuguagcucgggaa....................   1        0    seq
...........................................................augaagcacuguagcAc.........................   1        1    seq
...........................................................augaagcacuguagcucgggaa....................   1        0    seq
```
